# Supplementary material for: Candidate genes for shell colour polymorphism in Cepaea nemoralis
Source: PeerJ. 2017 Sep 18;5:e3715. doi: 10.7717/peerj.3715 (PMC5607911; doi:10.7717/peerj.3715)
Supplement: Supplemental Information 1 [file peerj-05-3715-s001.docx]

Supplementary material to:

**Candidate genes for shell colour polymorphism in *Cepaea nemoralis***

Jesse Kerkvliet, Tjalf de Boer, Menno Schilthuizen, Ken Kraaijeveld

Data S1-3 are provided as separate files.


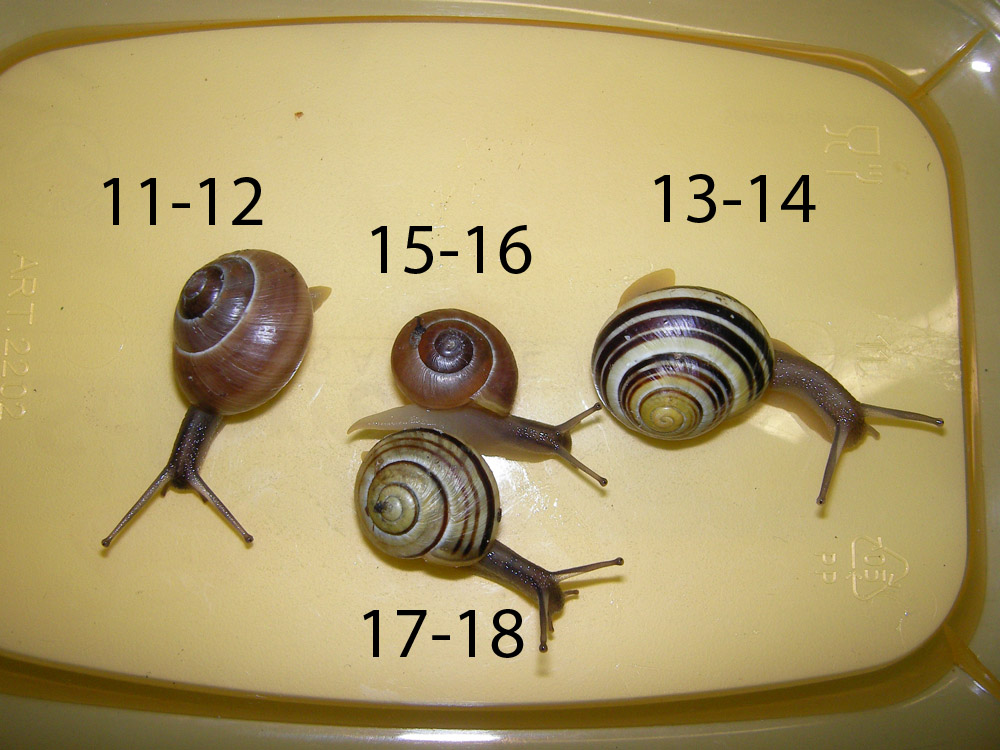


**Figure S1** Snails used in this study. Numbers correspond to those in Table 1.


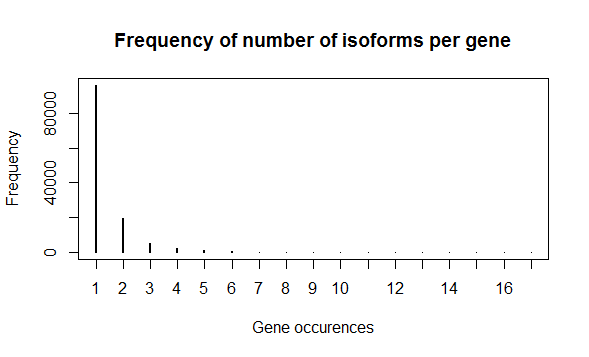


**Figure S2** Frequency distribution of the number of isoforms per gene.

**Table S1** Overview of the alignment statistics for each sample.

| Sample | Reads aligned at least once | Properly paired reads |
| --- | --- | --- |
| 11 | 38,337,898 (53.6%) | 33,244,620 (46.4%) |
| 12 | 52,000,948 (72.6%) | 45,560,146 (63.6%) |
| 13 | 50,780,782 (65.9%) | 44,080,380 (57.2%) |
| 14 | 47,651,676 (71,4%) | 41,350,714 (62.0%) |
| 15 | 47,852,396 (74.9%) | 42,796,990 (67.0%) |
| 16 | 54,298,143 (78.1%) | 49,089,688 (70.6%) |
| 17 | 91,448,178 (75.1%) | 82,673,624 (67.9%) |
| 18 | 106,823,206 (76.6%) | 96,221,182 (69.0%) |

**Table S2** Number of SNPS after filtering on sequence depth (>10) for each sample.

| Sample | SNPs |
| --- | --- |
| 11 | 81,233 |
| 12 | 132,825 |
| 13 | 135,232 |
| 14 | 117,458 |
| 15 | 155,511 |
| 16 | 98,933 |
| 17 | 200,068 |
| 18 | 155,360 |
| Total | 1,076619 |
| Total (unique) | 461,799 |
| Total (filtered) | 73,817 |

**Table S3** Annotations and predicted SNP effects. For each transcript, we report only the best hit based on e-value.

| **Contig name** | **Functional annotationKolom2** | **SNPEff effect** |
| --- | --- | --- |
| c264073_g1_i1 | Camelus bactrianus mucin-2-like | frameshift_variant&stop_gained |
|  |  | inframe_insertion |
| c280576_g1_i1 | Biomphalaria glabrata glycine and methionine-rich –like | synonymous_variant |
| c280925_g1_i2 | Parasteatoda tepidariorum keratin-associated 6-2-like | synonymous_variant |
| c321814_g1_i1 | Euhadra herklotsi mRNA for Dermatopontin1 | intergenic_region |
| c323639_g1_i1 | Aplysia californica multiple coagulation factor deficiency | synonymous_variant |
|  |  | 5_prime_UTR_variant |
| c344946_g1_i1 | Physa acuta G-type lysozyme mRNA, complete cds | missense_variant |
| c348138_g1_i1 | Biomphalaria glabrata annexin A7-like | chromosome_number_variation |
| c350256_g1_i1 | Anas platyrhynchos BPI fold-containing family B member 3 | synonymous_variant |
| c354311_g1_i1 | Biomphalaria glabrata sorting nexin-5-like | 5_prime_UTR_variant |
| c355427_g1_i1 | Euhadra herklotsi mRNA for Dermatopontin1 | stop_gained |
|  |  | missense_variant |
| c356371_g1_i1 | Aplysia californica tyramine beta-hydroxylase-like | missense_variant |
| c357948_g1_i2 | Aplysia californica nucleoside-diphosphatase | missense_variant |
| c358175_g7_i1 | AF109924Helix pomatia sulfatase 1 precursor | intergenic_region |
| c358705_g4_i1 | Aplysia californica carbonic anhydrase 1-like | stop_gained |
|  |  | missense_variant |
| c358903_g6_i2 | Aplysia californica toll-like receptor 3 | 5_prime_UTR_variant |
| c360655_g1_i2 | AF109924Helix pomatia sulfatase 1 precursor | disruptive_inframe_insertion |
|  |  | missense_variant |
|  |  | synonymous_variant |
|  |  | 5_prime_UTR_premature_start_codon_gain_variant |
| c360728_g7_i2 | Aplysia californica UPF0462 C4orf33 homolog | missense_variant |
| c361453_g1_i1 | Biomphalaria glabrata peroxidasin-like | 3_prime_UTR_variant |
|  |  | missense_variant |
| c361590_g1_i1 | Aplysia californica aquaporin-4-like | missense_variant |
|  |  | synonymous_variant |
| c362006_g8_i2 | Helix pomatia Cd-specific metallothionein gene | intergenic_region |
| c362358_g3_i1 | AF109924Helix pomatia sulfatase 1 precursor | intergenic_region |
| c362887_g2_i1 | AF109924Helix pomatia sulfatase 1 precursor | 3_prime_UTR_variant |
| c363384_g6_i1 | Helix pomatia Cd-specific metallothionein gene | intergenic_region |
| c363875_g1_i3 | Lepisosteus oculatus ficolin-2-like | missense_variant |
| c365069_g1_i1 | Biomphalaria glabrata tyrosinase | 5_prime_UTR_variant |
| c365850_g1_i1 | Biomphalaria glabrata formin BNR1-like | missense_variant |
|  |  | synonymous_variant |
| c366293_g1_i1 | Biomphalaria glabrata mucin-2-like | missense_variant |
| c367319_g1_i1 | Aplysia californica sodium-coupled monocarboxylate transporter | 5_prime_UTR_variant |
| c368154_g1_i1 | Lethenteron camtschaticum clone BAC 238J5 homeobox Hox-delta3 (Hox-delta3) and homeobox Hox-delta2 (Hox-delta2) genes | missense_variant |
|  |  | 3_prime_UTR_variant |
| c368572_g1_i1 | Aplysia californica epithelial splicing regulatory 1-like | synonymous_variant |
| c368766_g1_i1 | Aplysia californica mesenchyme-specific cell surface glyco -like | missense_variant |
| c369092_g1_i1 | Biomphalaria glabrata perivitellin-2 67 kDa subunit-like | synonymous_variant |
|  |  | missense_variant |
| c369237_g1_i2 | Biomphalaria glabrata amine oxidase | synonymous_variant |
| c369245_g1_i1 | Helix pomatia Cd-specific metallothionein gene | 3_prime_UTR_variant |
|  |  | synonymous_variant |
|  |  | missense_variant |
| c369506_g2_i1 | Aplysia californica solute carrier family 2, facilitated glucose transporter member | 5_prime_UTR_premature_start_codon_gain_variant |
|  |  | 5_prime_UTR_variant |
| c369765_g4_i2 | Biomphalaria glabrata ferric-chelate reductase 1-like | synonymous_variant |
| c369942_g2_i3 | Aplysia californica ATP-binding cassette sub-family G member 2-like | missense_variant |
| c371799_g2_i1 | Biomphalaria glabrata sushi, von Willebrand factor type A, EGF and pentraxin domain-containing 1-like | synonymous_variant |

**Table S4** Transcripts with hits on the four RAD tags near the supergene.

| RAD tag ID | Contig | Blastn annotation |
| --- | --- | --- |
| Cne_RAD02 | c364559_g2_i4 | None |
| Cne_RAD02 | c366252_g1_i1 | None |
| Cne_RAD02 | c366252_g1_i2 | None |
| Cne_RAD02 | c366252_g1_i6 | None |
| Cne_RAD08 | c365766_g3_i1 | *Helix pomatia* methallothionein |
| Cne_RAD08 | c363423_g3_i1 | *Helix pomatia* methallothionein |
| Cne_RAD08 | c337572_g1_i1 | *Helix pomatia* methallothionein |
| Cne_RAD10 | c360230_g9_i1 | *Helix pomatia* methallothionein |
| Cne_RAD10 | c360230_g8_i1 | *Helix pomatia* methallothionein |
| Cne_RAD10 | c367196_g3_i3 | *Helix pomatia* methallothionein |
| Cne_RAD10 | c367196_g3_i1 | *Helix pomatia* methallothionein |
| Cne_RAD10 | c325228_g1_i1 | *Helix pomatia* methallothionein |
